# TrpC5 silencing reduces cell invasion and migration and enhances radiosensitivity in papillary thyroid carcinoma cells

**DOI:** 10.3389/fcell.2026.1705616

**Published:** 2026-02-13

**Authors:** Jing Yang, Zijiang Sang, Haibing Yang, Lvzhou Cao, Jinxia Chen, Jiaxin Yang, Rixiang Gong, Zhihui Li, Yanping Gong

**Affiliations:** 1 Division of Thyroid Surgery, Department of General Surgery, West China Hospital, Sichuan University, Chengdu, Sichuan, China; 2 Laboratory of Thyroid and Parathyroid Diseases, Frontiers Science Center for Disease-Related Molecular Network, West China Hospital, Sichuan University, Chengdu, Sichuan, China; 3 Department of Breast and Thyroid Surgery, People’s Hospital of Qinghai Province, Qinghai, China; 4 Division of Thyroid Surgery, Department of General Surgery, West China Tianfu Hospital, Sichuan University, Chengdu, Sichuan, China

**Keywords:** DNA damage, invasion, papillary thyroid carcinoma, radiosensitivity, transient receptor potential channel C5

## Abstract

**Introduction:**

Carcinoma (PTC) is the most common malignant tumor derived from thyroid follicular cells and represents the most common pathological type of thyroid malignancy. Cancer metastasis and radiosensitivity are important factors that limit the treatment of PTC. This study aimed to investigate the regulatory effects of transient receptor potential channel C5 (TrpC5) on the proliferation, invasion, migration, and radiosensitivity of PTC.

**Methods:**

Human papillary thyroid carcinoma cell lines TPC-1 and B-CPAP were transfected with TrpC5 siRNA, pcDNA-TrpC5, or their corresponding negative control. PTC cells were stimulated by radiotherapy.

**Results:**

The results showed that TrpC5 silencing weakened the proliferation, invasion, and migration of PTC cells, whereas TrpC5 overexpression promoted these cellular behaviors. Moreover, TrpC5 expression was progressively upregulated in PTC cells following exposure to irradiation (IR). TrpC5 silencing enhanced radiosensitivity of TPC-1 and B-CPAP cells. In addition, TrpC5 silencing enhanced the expression of DNA damage-related proteins p-ATM, p-CHK, and γH2AX in PTC cells under IR treatment. Overall, TrpC5 silencing weakened cell invasion and migration and enhanced the radiosensitivity of PTC cells.

**Conclusion:**

These findings suggest that TrpC5 may serve as a potential therapeutic target for PTC and warrant further investigation *in vivo*.

## Background

Papillary thyroid carcinoma (PTC) is a differentiated malignant tumor originating from thyroid follicular epithelial cells and is the most common pathological type of thyroid carcinoma (Stephen et al., 2020; Kola et al., 2021). The incidence of thyroid cancer has been increasing in recent years, and PTC progresses faster than other types (Kola et al., 2021). Patients with PTC, which poses a serious threat to health, are increasingly younger, and the disease is more prevalent in female individuals (Liu et al., 2019). PTC often does not present typical clinical symptoms, except for slow-growing thyroid nodules; therefore, it is easily misdiagnosed, potentially causing patients to miss the optimal treatment period (Coca-Pelaz et al., 2020). Although the overall prognosis of PTC is generally good, some cases are highly aggressive, exhibiting early tumor invasion and lymph node metastasis, and are prone to recurrence and metastasis after surgery (Yu et al., 2020). Therefore, an urgent issue in both clinical and basic research on PTC is to identify related target molecules and molecular mechanisms involved in its occurrence and development, exploring new and effective PTC molecular markers for diagnosis, treatment, and prognosis.

Transient receptor potential channel C5 (TrpC5) is a member of the transient receptor potential (TRP) non-selective cation channel family and plays an important role in many physiological processes. It is associated with cardiovascular disease, central nervous system disease, and cancers. It has been demonstrated that TrpC5 is associated with the progression and treatment of a variety of cancers, including colorectal cancer, breast cancer, and cancer of the nervous system. TrpC5 has been suggested as a promising therapeutic target for cancer due to its significance in the development and therapy of cancers. It has been demonstrated in the previous study that there is a marked association between high TrpC5 expression and poor prognosis in patients with radical colorectal cancer (Cai et al., 2021). Meanwhile, in colorectal cancer, TrpC5 regulated cell differentiation via Ca^2+^/Wnt5a signaling (Chen et al., 2017a). Overexpression of TrpC5 promoted tumor metastasis via the HIF-1α-twist signaling pathway in colon cancer (Chen et al., 2017b). TrpC5-induced autophagy promotes drug resistance in breast cancer via the CaMKKβ/AMPKα/mTOR pathway (Zhang et al., 2017). However, the role of TrpC5 in regulating the malignant biological behavior and radiosensitivity of PTC has not been reported.

In this study, we demonstrated that TrpC5 expression was upregulated in irradiation (IR)-treated PTC cells. Functional analysis revealed that TrpC5 silencing inhibited the proliferation, migration, and invasion of PTC cells. TrpC5 overexpression showed the opposite results. Furthermore, TrpC5 enhanced the radiosensitivity of PTC cells.

## Methods

### Cell culture and transfection

Human papillary thyroid carcinoma cell lines TPC-1 (CL-0643) and B-CPAP (CL-0575) were obtained from Procell (Wuhan, China). After centrifugation at 1000 × *g* for 10 min, cells were cultured in DMEM complete medium (Gibco BRL, United States) supplemented with 10% fetal bovine serum (FBS, Gibco BR L, United States) at 37 °C in a humidified atmosphere containing 5% CO_2_. For gene silencing, two specific siRNAs targeting TrpC5 (TrpC5-siRNA-1 and TrpC5-siRNA-2) and a negative control siRNA (NC siRNA) were used. The siRNA sequences were as follows: TrpC5-siRNA-1: (5′- GGA​CAG​GAA​GAA​CAA​GUU​ACA-3′) and TrpC5-siRNA-2: (5′- GGA​AGU​UAU​CAG​GAA​UUU​AGU-3′). A non-targeting scrambled siRNA (siRNA-NC) was used as the negative control (5′-UUC​UCC​GAA​CGU​GUC​ACG​U-3′). For TrpC5 overexpression, the pcDNA3.1 vector containing the full-length human TrpC5 cDNA (pcDNA-TrpC5) and the empty vector (pcDNA-NC) were used. Lipofectamine® 2000 transfection reagent (Invitrogen, Grand Island, NY) was used for the transient transfection of the above-mentioned constructs into TPC-1 and B-CPAP cells (1.0 × 10^5^ cells/well), according to the manufacturer’s instructions. The knockdown and overexpression efficiency were validated at both the mRNA and protein levels using RT-qPCR and Western blot analysis, respectively, at 48 h post-transfection.

### Irradiation exposure

An irradiation apparatus (2100C/D, VARIAN, Palo Alto, CA, United States) generated a 6 MV photon beam that was used to irradiate exponentially growing PTC cells at room temperature. To investigate the correlation between TrpC5 expression and irradiation time, cells were exposed to a single dose of 2 Gy and harvested at 0, 4, 8, 12, 16, 20, and 24 h post-irradiation for RT-qPCR and Western blot analysis (Figure 2). For the radiosensitivity assays (Figure 3), cells transfected with siRNA-NC or siRNA-TrpC5 were irradiated with varying doses (0, 2, 4, 6, and 8 Gy) using the same apparatus. For all other experiments involving apoptosis and DNA damage protein detection, a single dose of 2 Gy was applied.

### Real-time fluorescence quantitative polymerase chain reaction

The mRNA expression of TrpC5 was evaluated using real-time fluorescence quantitative polymerase chain reaction (RT-qPCR). For gene analysis, equal amounts of cDNA were added to a reaction mixture containing gene-specific forward and reverse primers, deoxynucleotides, Taq DNA polymerase, and SYBR Green (Bio-Rad, Hercules, CA). Quantification of cDNA was performed by monitoring the increase in SYBR Green fluorescence during the exponential phase of amplification using an RT-qPCR system (Bio-Rad, Hercules, CA) and determining the PCR cycle at which the amplified product exceeded a defined threshold.

### Western blot analysis

Cell lysis solution for TPC-1 and B-CPAP cells was prepared using RIPA buffer (Santa Cruz Biotechnology, Dallas, TX) to extract total proteins. Protein concentration was determined using the Bicinchoninic Acid (BCA) Protein Assay Kit (Pierce, Rockford, IL). Thirty micrograms of total cellular protein were subjected to SDS-PAGE, followed by electrophoretic transfer to nitrocellulose. Filters were probed with a primary antibody, followed by an HRP-conjugated anti-rabbit-IgG secondary antibody, and then developed using the ECL system (Amersham, Piscataway, NJ). Densitometric analysis was performed using Scion Image data analysis software (Scion Corporation, Frederick, MD). The corresponding protein antibodies were as follows: TrpC5 (Abcam, ab240872; 1/1,000), p-ATM (Abcam, ab81292; 1/50,000), p-CHK (CHAT, ABclonal, A21009; 1/1,000), γH2AX (Abcam, ab81299; 1/5,000), and β-actin (Boster, BM0627; 1/1,000).

### Flow cytometry assay

The apoptosis of TPC-1 and B-CPAP cells was analyzed using Annexin V, according to the manufacturer’s protocol. In brief, the cells were washed with PBS (Invitrogen, Carlsbad, CA, United States), and the cell concentration was adjusted to 1.0 × 10^6^ cells/mL. The cells were subsequently suspended in a 150-μL buffering solution. Subsequently, the cells were stained with 10 μg/mL Annexin V-FITC and 5 μL PI at 4 °C for 20 min in the dark. Apoptotic cells were subsequently analyzed using a BD FACSCelesta™ Flow Cytometer (Becton, Dickinson, and Company).

### CCK-8 assay

TPC-1 and B-CPAP cells were digested and seeded in 96-well plates (1.0 × 10^9^ cells/well). An aliquot of 100 μL of DMEM containing 10% FBS and 10 μL of cell counting kit-8 (CCK-8) solution was added to each well, and the plates were incubated for 0.5–2 h in a cell incubator. Absorbance was then measured at 450 nm.

### Clone formation assay

TPC-1 and B-CPAP cells (1.0 × 10^3^ cells/well) were seeded in six-well plates and incubated at 37 °C with 5% CO_2_ for 2 weeks. Following incubation, the cells were fixed in formaldehyde and then stained with 0.1% crystal violet for 5 min at room temperature. The number of colonies (>50 cells) was counted using an optical inverted microscope (Olympus Corporation).

### Wound-healing assay

TPC-1 and B-CPAP cells were cultured in 96-well plates until they reached confluence. Cells monolayers were scraped with a 200-μL pipette tip. The fresh medium was replaced, and cells were grown continuously. Then, the medium was used to gently wash the wells of the plate to remove exfoliated cells. The range between the two edges of the wound was calculated. In addition, multiple visual fields were selected for the observation of each hole. After 24 h, the distance of the wound channel is supposed to be surveyed again. Scratch areas were quantified using ImageJ software.

### Transwell assay

A total of 1 × 10^5^ TPC-1 and B-CPAP cells/mL were resuspended in DMEM, and 200 µL of cell suspension/well was plated into the upper chambers of 24-well Transwell plates. The lower chambers were filled with 600 μL of DMEM supplemented with 10% FBS (HyClone; Cytiva). The cells were then placed in an incubator (5% CO_2_ and 37 °C) for 48 h. Finally, the cells were fixed in 4% paraformaldehyde for 20 min and stained with 0.1% crystal violet for 15 min. An inverted microscope (Olympus Corporation) was used to observe the number of cells in five randomly selected fields of view.

### Statistical analysis

All data are presented as the mean ± standard deviation (SD). The results were obtained from three independent experiments (biological replicates, n = 3). Within each independent experiment, measurements were performed with six technical replicates (n = 6) for assays such as CCK-8. Statistical significance between multiple groups was determined using one-way analysis of variance (ANOVA), followed by Tukey’s *post hoc* test for multiple comparisons. A difference of *p* < 0.05 was defined as statistically significant.

## Results

### TrpC5 regulated the proliferation, migration, and invasion of PTC cells

**FIGURE 1:**
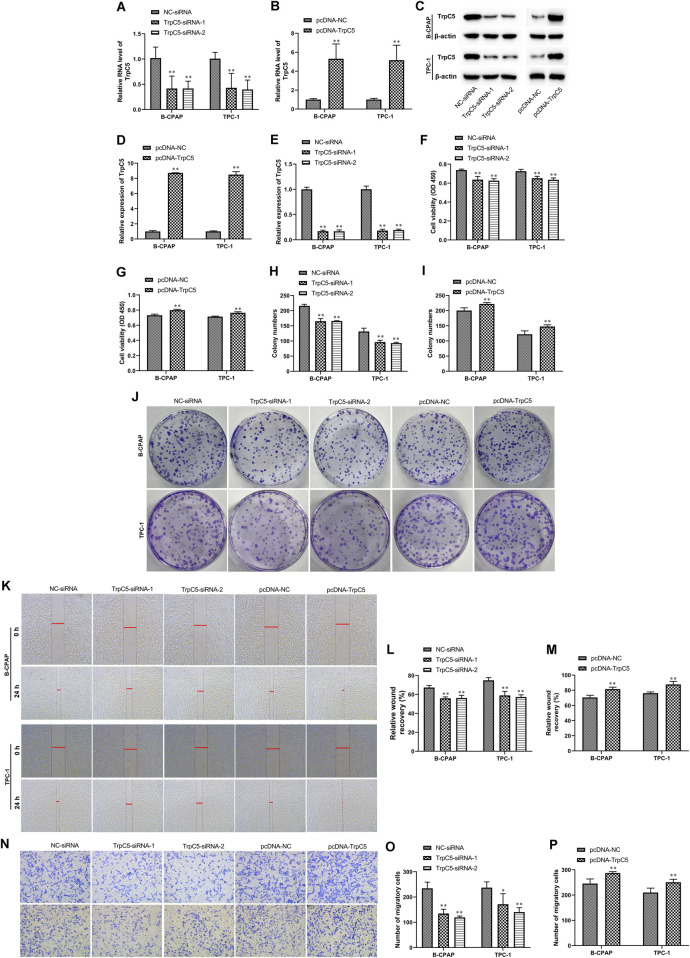
TrpC5 regulated PTC cell proliferation. **(A,B)** The mRNA level of TrpC5 in TPC-1 and B-CPAP cells was evaluated using RT-qPCR. **(C–E)** The protein level of TrpC5 in TPC-1 and B-CPAP cells was determined using Western blot analysis. **(F–J)** The effect of TrpC5 silencing or overexpression on the growth of TPC-1 and B-CPAP cells was detected using CCK-8 and colony formation assays. **(K–M)** The effect of TrpC5 silencing or overexpression on TPC-1 and B-CPAP cell invasion was detected by Wound-healing assay. **(N–P)** Transwell analysis was performed in TPC-1 and B-CPAP cells transfected with TrpC5-siRNA or pcDNA-TrpC5. Values represent the mean ± SD. ^**^ *p* < 0.01 vs. NC-siRNA or pcDNA-NC.

siRNA was constructed to reduce TrpC5 expression. As shown in Figures 1A,C,E, TrpC5 expression was decreased effectively by TrpC5-siRNA-1 and TrpC5-siRNA-2 in TPC-1 and B-CPAP cells. Meanwhile, TrpC5 expression was increased effectively by pcDNA-TrpC5 (Figures 1B–D). We demonstrated that TrpC5 silencing inhibited the viability of PTC cells (Figures 1F,H,J). TrpC5 overexpression promoted the viability of PTC cells (Figures 1G,I,J). In addition, silencing of TrpC5 weakened cell invasion and migration of PTC cells (Figures 1K–P). TrpC5 overexpression stimulated cell invasion and migration of PTC cells (Figures 1K–P).

### TrpC5 expression was progressively upregulated in PTC cells following exposure to IR

**FIGURE 2:**
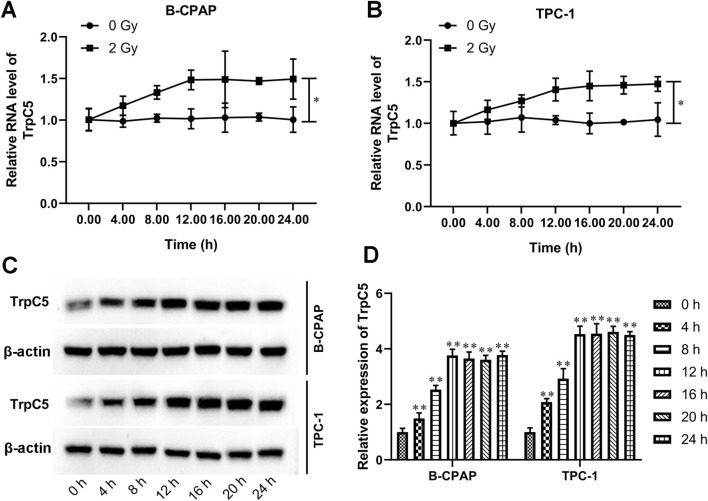
TrpC5 expression was progressively upregulated in PTC cells following exposure to IR. **(A,B)** TPC-1 and B-CPAP cells were treated with 2 Gy IR at different time points, followed by qRT-PCR detection of the mRNA expression patterns of TrpC5 every 4 h for 24 h. **(C,D)** The TrpC5 protein level every 4 h for 24 h was measured using Western blot after exposure to 2 Gy in TPC-1 and B-CPAP cells. Values represent the mean ± SD. ^*^ *p* < 0.05 vs. 2 Gy; ^**^ *p* < 0.01 vs. 0 h.

Subsequently, the effect of irradiation on TrpC5 expression was assessed every 4 h for 24 h after exposure to 2 Gy irradiation in PTC cells. As shown in Figures 2A,B, compared with cells without radiation treatment, the TrpC5 mRNA level was markedly induced after irradiation treatment in TPC-1 and B-CPAP cells. Moreover, Western blot analysis showed that the TrpC5 protein level was induced after irradiation treatment in TPC-1 and B-CPAP cells (Figures 2C,D).

### TrpC5 silencing enhanced the radiosensitivity of PTC cells

**FIGURE 3:**
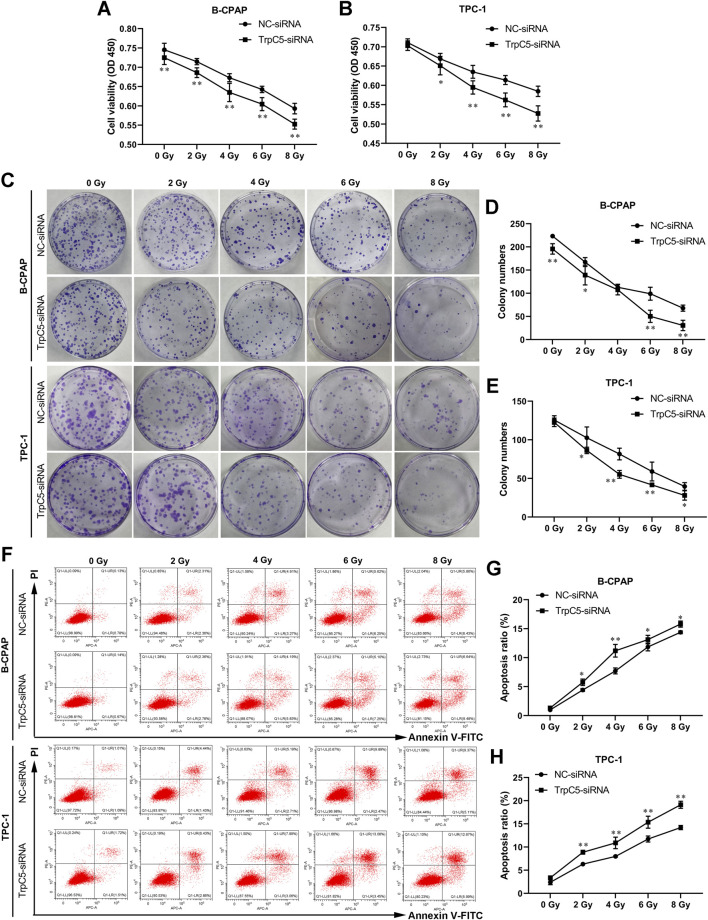
TrpC5 silencing enhanced radiosensitivity of PTC cells. TPC-1 and B-CPAP cells were transfected with NC-siRNA or TrpC5-siRNA for 48 h. **(A–E)** The transfected TPC-1 and B-CPAP cells were exposed to different doses of IR (0, 2, 4, 6, and 8 Gy), and survival fraction was detected using CCK-8 and colony formation assays. **(F–H)** The transfected TPC-1 and B-CPAP cells were treated with IR (0, 2, 4, 6, and 8 Gy), followed by the measurement of the cell apoptosis rate using flow cytometry analysis. Values represent the mean ± SD. ^*^ *p* < 0.05 and ^**^ *p* < 0.01 vs. NC-siRNA.

Subsequently, experiments were performed to investigate the effect of TrpC5 on radiosensitivity in PTC cells. CCK-8 and colony formation assays demonstrated that TrpC5 silencing markedly weakened the survival fraction of TPC-1 and B-CPAP cells, indicating that TrpC5 silencing enhanced radiosensitivity in PTC cells (Figures 3A–E). Flow cytometry analysis revealed that TrpC5 silencing enhanced apoptosis in TPC-1 and B-CPAP cells following IR treatment (Figures 3F–H).

### TrpC5 silencing enhanced DNA damage-related proteins in PTC cells under IR treatment

**FIGURE 4:**
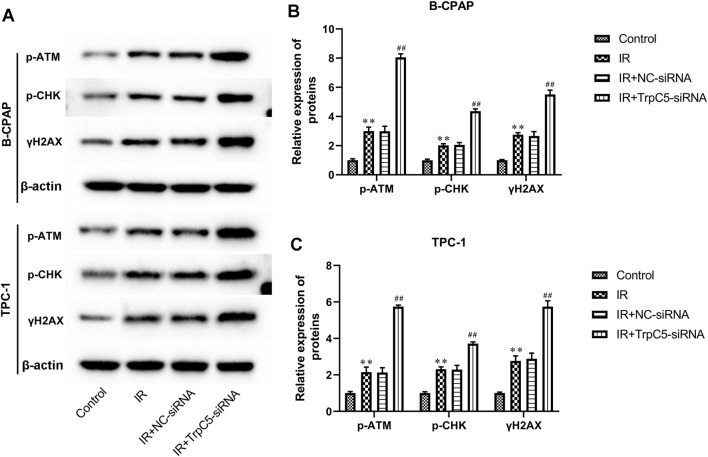
TrpC5 silencing enhanced DNA damage-related proteins in PTC cells under IR treatment. TPC-1 and B-CPAP cells were transfected with NC-siRNA or TrpC5-siRNA for 48 h. Then, the transfected TPC-1 and B-CPAP cells were treated with 2 Gy. **(A–C)** The protein expressions of p-ATM, p-CHK, and γH2AX in TPC-1 and B-CPAP cells were evaluated using Western blot analysis. Values represent the mean ± SD. **p > 0.01 vs. control; ^##^ *p* > 0.01 vs. IR + NC-siRNA.

As shown in Figures 4A–C, IR treatment increased the expressions of DNA damage-related proteins p-ATM, p-CHK, and γH2AX in TPC-1 and B-CPAP cells. Meanwhile, TrpC5 silencing markedly enhanced p-ATM, p-CHK, and γH2AX expressions in TPC-1 and B-CPAP cells under IR treatment (Figures 4A–C).

## Discussion

TrpC5 plays a vital function in regulating the chemotherapy sensitivity of cancer cells. This study has reported that TrpC5 may be the cause of the resistance of breast cancer to chemotherapy drugs (Ma et al., 2012). P-glycoprotein (P-gp), also known as multidrug resistance protein 1, is a multidrug efflux transporter that can remove exogenous substances from the cell (Pokharel et al., 2017). Therefore, after overexpression of P-gp, malignant tumor cells become insensitive to chemotherapy drugs such as doxorubicin, vincristine, paclitaxel, and anthracyclines (Pokharel et al., 2017). There was evidence that in breast cancer Adriamycin-resistant cell lines, TrpC5 was abnormally highly expressed and regulated the expression of P-gp through the Ca^2+^-dependent transcription factor NFATC3, thereby affecting the sensitivity of cells to Adriamycin (Ma et al., 2012). Furthermore, breast cancer drug resistance is promoted by TrpC5-induced autophagy via the CaMKKβ/AMPKα/mTOR pathway (Zhang et al., 2017). TrpC5 upregulated temozolomide (TMZ) resistance via the CaMKKβ/AMPKα/mTOR pathway in glioma chemotherapy (Zou et al., 2019). Many biological methods can regulate the radiosensitivity of PTC. Sinomenine hydrochloride markedly enhanced the radiosensitivity of HTori-3 and PTC cells, which was associated with the downregulation of the Bcl-2/Bax protein ratio and upregulation of Fas, p21, p-ATM, p-CHK1, p-CHK2, and p53 protein expression (Zhao et al., 2021). *In vitro* and *in vivo*, adenovirus-mediated siRNA targeting NOB1 inhibited PTC tumor growth and enhanced radiosensitivity (Meng et al., 2014). Moreover, autophagy induction with RAD001 enhanced chemosensitivity and radiosensitivity through Met inhibition in PTC (Lin et al., 2010). However, the modulating effect of TrpC5 on tumor radiotherapy sensitivity has not been studied. Our data showed that TrpC5 silencing enhanced the radiosensitivity of PTC cells.

The DNA damage response (DDR) is a precise signaling cascade system that detects DNA damage and determines the fate of affected cells. It is an important area of research in tumor biology, particularly due to its key role in the regulation of tumor radiosensitivity (Huang and Zhou, 2020). An important reason for the resistance of tumor cells to radiotherapy is that cancer cells can recognize DNA damage induced by ionizing radiation and repair it by activating various pathways (Willers et al., 2015; Morgan and Lawrence, 2015). Cancer cells exhibit high DNA repair activity, which contributes to their pronounced resistance to radiotherapy (Ratnayake et al., 2018). Studies have shown that targeting DNA damage repair can effectively improve the sensitivity of cancer to radiotherapy (Luo and Leverson, 2005; Jeggo, 2010). As the core protein kinases of DNA damage response, ATM and ATR can activate downstream DNA damage response-related molecules such as CHK1, CHK2, and p53, thereby regulating various cellular responses. Ionizing radiation-induced rapid phosphorylation of the nucleosomal histone H2AX at serine 139 (γH2AX) at DNA double-strand break sites occurs and can be visualized as a fluorescent focus (Löbrich et al., 2010). The γH2AX focus is formed within a few minutes after the ionizing radiation produces a double-strand break and disappears after a few hours due to the action of DNA repair enzymes (Löbrich et al., 2010). Paclitaxel increased p-ATM and γH2AX levels and enhanced radiosensitivity in PTC by promoting p53-dependent apoptosis (Wu et al., 2021). Canagliflozin (an SGLT2 inhibitor) increased γH2AX expression and activated the DNA damage response signaling pathway ATM/CHK2 in PTC cells (Wang et al., 2022).

Our findings offer a potential mechanistic explanation for this biological process. We show that silencing of TrpC5 markedly enhances IR-induced phosphorylation of ATM and CHK2, along with the formation of γH2AX foci, indicative of an amplified DDR. These results suggest that TrpC5 may act as a negative regulator within the DDR signaling cascade. One possible mechanism is that TrpC5-mediated Ca^2+^ influx modulates the activity or recruitment of key kinases involved in the DDR pathway. This is plausible given the established role of calcium as a secondary messenger in numerous signaling pathways, including those that can influence cellular stress responses (Kumaş-Kulualp and Gepdiremen, 2023). Alternatively, TrpC5 may interact with proteins that inhibit the initiation of DDR signaling. Although a direct link between TrpC5 and DDR has not been reported, other ion channels have been implicated in regulating DNA repair processes (Prevarskaya et al., 2018). Consequently, suppression of TrpC5 could impair efficient DNA repair by attenuating the DDR, resulting in the accumulation of lethal DNA lesions and ultimately increasing cellular radiosensitivity—findings supported by our clonogenic survival and apoptosis assays. This previously uncharacterized role of TrpC5 in negatively regulating the DDR provides a molecular basis for its contribution to radioresistance.

The ability of tumor cells to metastasize is an important hallmark of malignant tumors, and invasion is the first and most important step in the metastasis process (Wan et al., 2019). The ability of tumor cells to invade and migrate underlies tumor metastasis (Duff and Long, 2017; van de Merbel et al., 2018). In recent studies, certain voltage-independent Ca^2+^ channels, especially subtypes from the Orai and the TRP families, have been implicated in mesenchymal migration and metastasis formation (Déliot and Constantin, 2015). TrpC3 was reported to regulate melanoma proliferation and migration (Oda et al., 2017). Through inhibition of the PI3K/AKT pathway, TrpC1 inhibited the proliferation and migration of estrogen receptor-positive breast cancer (Liu, 2024). Through modulation of Orai1 and Orai3 surface exposure, TrpC6 channels are essential for the proliferation, migration, and invasion of breast cancer cells (Jardin et al., 2018). TrpC1 promoted tumor growth and metastasis of colorectal cancer by activating the CaM-mediated PI3K/AKT signaling axis (Sun et al., 2021). Importantly, overexpression of TrpC5 promoted tumor metastasis via the HIF-1α-twist signaling pathway in colon cancer. This study suggested that TrpC5 overexpression stimulated cell invasion and migration of PTC cells, implying that TrpC5 played a promotive role in the development of PTC.

### Study limitations

This study did not involve any animal experiments. In future studies, we will further investigate the therapeutic effect of TrpC5 on PTC using animal models.

## Conclusion

Taken together, we found that TrpC5 overexpression stimulated the proliferation, invasion, and migration of PTC cells. Moreover, TrpC5 silencing enhanced radiosensitivity and increased the expression of DNA damage-related proteins in PTC cells. Our *in vitro* data indicate that TrpC5 could be a promising molecular target for the treatment of PTC. Future studies using animal models are necessary to validate these findings and explore their clinical translational potential. This study may provide a valid molecular target for the clinical treatment of PTC.

## Data Availability

The original contributions presented in the study are included in the article/Supplementary Material; further inquiries can be directed to the corresponding author.
